# Quantitative Analysis of Arsenic- and Sucrose-Induced Liver Collagen Remodeling Using Machine Learning on Second-Harmonic Generation Microscopy Images

**DOI:** 10.3390/cells15030214

**Published:** 2026-01-23

**Authors:** Mónica Maldonado-Terrón, Julio César Guerrero-Lara, Rodrigo Felipe-Elizarraras, C. Mateo Frausto-Avila, Jose Pablo Manriquez-Amavizca, Myrian Velasco, Zeferino Ibarra Borja, Héctor Cruz-Ramírez, Ana Leonor Rivera, Marcia Hiriart, Mario Alan Quiroz-Juárez, Alfred B. U’Ren

**Affiliations:** 1 Instituto de Ciencias Nucleares, Universidad Nacional Autónoma de México, Cto. Exterior S/N, C.U., Coyoacán, Ciudad de México 04510, Mexico; monica.maldonado@correo.nucleares.unam.mx (M.M.-T.); juliocgl2@ciencias.unam.mx (J.C.G.-L.); zeferino.ibarra@correo.nucleares.unam.mx (Z.I.B.); hector.cruz@correo.nucleares.unam.mx (H.C.-R.); ana.rivera@nucleares.unam.mx (A.L.R.); 2 Departamento de Física, Cinvestav, Av Instituto Politécnico Nacional 2508, La Laguna Ticoman, Gustavo A. Madero, Ciudad de México 07360, Mexico; rodrigo.felipe@cinvestav.mx; 3 Centro de Física Aplicada y Tecnología Avanzada, Universidad Nacional Autónoma de México, Boulevard Juriquilla 3001, Juriquilla, Querétaro 76230, Mexico; mfrausto@fata.unam.mx (C.M.F.-A.); maqj@fata.unam.mx (M.A.Q.-J.); 4 Tecnológico de Monterrey, Calle Epigmenio González 500, Fraccionamiento Vista 2000, Querétaro 76130, Mexico; a01708548@tec.mx; 5 Department of Cognitive Neurosciences, Instituto de Fisiología Celular, Universidad Nacional Autónoma de México, Cto. Exterior s/n, C.U., Coyoacán, Ciudad de México 04510, Mexico; mvelasco@ifc.unam.mx (M.V.); mhiriart@ifc.unam.mx (M.H.)

**Keywords:** second-harmonic generation microscopy, liver fibrosis, non-alcoholic fatty liver disease (NAFLD), machine learning image classification, sucrose diet, arsenic diet

## Abstract

Non-alcoholic fatty liver disease (NAFLD) is a silent condition that can lead to fatal cirrhosis, with dietary factors playing a central role. The effect of various dietary interventions on male Wistar rats were evaluated in four diets: control, arsenic, sucrose, and arsenic–sucrose. SHG microscopy images from the right ventral lobe of the liver tissue were analyzed with a neural network trained to detect the presence or absence of collagen fibers, followed by the assessment of their orientation and angular distribution. Machine learning classification of SHG microscopy images revealed a marked increase in fibrosis risk with dietary interventions: <10% in controls, 24% with arsenic, 40% with sucrose, and 62% with combined arsenic–sucrose intake. Angular width distribution of collagen fibers narrowed dramatically across groups: 26° (control), 24° (arsenic), 15.7° (sucrose), and 2.8° (arsenic–sucrose). This analysis revealed four key statistical features for classifying the images according to the presence or absence of collagen fibers: (1) the percentage of pixels whose intensity is above the 15% noise threshold, (2) the Mean-to-Standard Deviation ratio (Mean/std), (3) the mode, and (4) the total intensity (sum). These results demonstrate that a diet rich in sucrose, particularly in combination with arsenic, constitutes a significant risk factor for liver collagen fiber remodeling.

## 1. Introduction

The causes of liver fibrosis are varied, including non-alcoholic fatty liver disease (NAFLD), alcohol consumption, some viral diseases, and diet [1]. NAFLD, the most common chronic liver disease worldwide, develops when excessive lipids are stored in liver cells. Although this condition was initially associated with overweight and obese individuals, it is now increasingly observed in patients with a normal body mass index (BMI). This dissociation clearly indicates that non-weight-related factors, such as dietary habits, are significant to the disease’s pathogenesis. NAFLD represents a spectrum of conditions that range from simple fatty liver to steatohepatitis, fibrosis, cirrhosis, and hepatocellular carcinoma [2].

The liver, the largest internal organ in adults, plays a critical role in metabolism, detoxification, and homeostasis [3]. The liver has a large regenerative capacity. However, it is also vulnerable to injuries that lead to collagen deposition and fibrosis. On the one hand, environmental factors, including exposure to sucrose and arsenic, have an impact on liver health. In recent decades, humanity has faced a surge in overweight and obesity, in part due to excess sucrose in the diet. On the other hand, arsenic is a toxic metalloid that originates from both natural and anthropogenic sources and may contaminate water sources and food. Daily arsenic exposure in human adults depends heavily on geographic location and diet, with dietary patterns and the quality of local water sources primarily mediating these effects. In European adults, the average daily arsenic intake is estimated to be between 0.03 and 0.33 µg/kg body weight [4]. Consequently, for an individual weighing 80 kg, this translates to a total daily exposure of 2.4 to 26.4 µg. In contrast, certain high-risk regions exhibit extreme exposure levels, with daily arsenic intakes reaching as high as 685 µg per person [5]. Arsenic consumption in the daily diet is associated with serious health risks, including metabolic disorders [6,7]. Both agents alter liver function and have been linked to insulin resistance through impaired translocation of GLUT4 in animal models [8,9].

In the past two decades, second-harmonic generation microscopy (SHG) has become a powerful tool in materials and biomedical sciences [10]. Its biomedical use began when collagen was identified as a strong SHG emitter approximately two decades ago [11]. As the most abundant structural protein in vertebrates, the organization and distribution of collagen serve as valuable biomarkers for various diseases [12]. Changes in collagen fiber orientation and organization have been associated with several pathological conditions, including pulmonary diseases [13], ocular disorders [14,15], tendinopathies, and cancers [16]. SHG microscopy enables the visualization and quantitative analysis of collagen fiber distribution, density, and orientation in healthy and diseased tissues [17,18]. However, traditional SHG imaging often fails to provide sufficiently detailed quantitative information to accurately assess disease severity. A major challenge is the tissue-specific nature of collagen characteristics, which limits the direct transferability of findings between tissues. Thus, independent optimization of experimental parameters is required for each tissue type.

Three key factors influence the optimization of the SHG signal:

- Experimental parameters: Wavelength and polarization strongly affect SHG intensity. While longer wavelengths (>1000 nm) improve penetration depth, intratissue scattering may reduce the usable signal [19]. Polarization dependence is significant; While parallel polarization may maximize the signal, elliptical and circular polarizations permit orientation-insensitive SHG generation [18,20].
- Biomechanical and biochemical parameters: Collagen density, fiber organization, and sample thickness impact SHG intensity. Diseases typically disrupt fiber alignment and modify the forward/backward (F/B) signal ratio [17,18,21,22,23].
- Computational tools: Computational approaches such as Monte Carlo modeling and machine learning are useful for quantifying SHG images, as they enable modeling of light–tissue interactions and support automated fiber analysis, noise reduction, and image classification for localized tissue health assessment [12,16,24,25].

SHG microscopy is based on SHG, a nonlinear, coherent optical process in which photons are generated at twice the frequency of the incident beam in media with nonzero second-order susceptibility [26]. In SHG, two photons at frequency ω combine to produce a photon at 2ω [27]. First demonstrated in bulk crystals in 1961, SHG has found applications in materials characterization [28], frequency conversion in pulsed lasers [29], and imaging of collagen-rich tissues [30]. Unlike two-photon excited fluorescence (TPEF), SHG is non-resonant, and the produced flux exhibits a quadratic dependence on the pump intensity [26,31]. While SHG in phase-matched crystals is highly directional and predominantly forward-propagating, in biological tissue, spatial variations in refractive index and nonlinear susceptibility, combined with scattering, result in both forward and backward emission [32].

In this study, we exploit the self-learning capabilities of machine learning algorithms to classify SHG images of liver tissue based on the presence of collagen. A neural network was specifically trained and tested to address the binary classification task of distinguishing collagen-positive from collagen-negative samples. To this end, a controlled experimental study was conducted to evaluate the effects of four dietary interventions—control (Group C), arsenic (Group A), sucrose (Group S), and arsenic–sucrose (Group AS) diets—on SHG signals in rat liver tissue. The trained model achieved high accuracy in classifying collagen presence and effectively captured the influence of dietary factors from the SHG images. Our findings indicate that the intake of sucrose and combined arsenic–sucrose mixtures represents a significant risk factor for liver fibrosis, as reflected by marked alterations in collagen fiber organization and angular distribution. In contrast, the control and arsenic-only diets exhibited a substantially lower risk.

## 2. Materials and Methods

**Figure 1:**
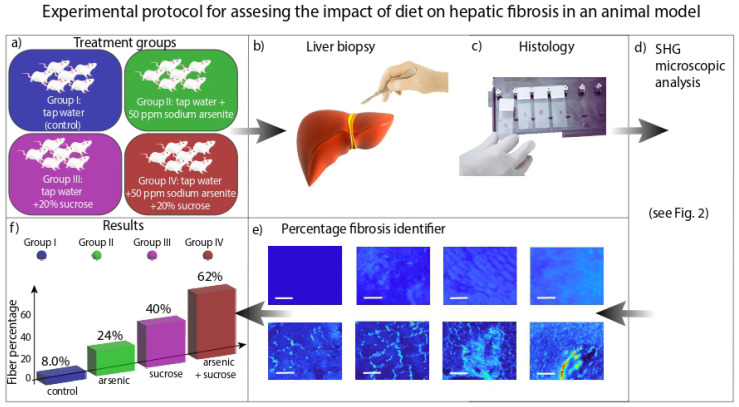
Diagram of the methodology: (**a**) define the treatment groups, (**b**,**c**) conduct a liver biopsy procedure and histology, (**d**) perform SHG microscopic analysis on the samples (low and high resolution), (**e**) identify the optimal features for classifying images into fibrotic and non-fibrotic, the scale bar represents 0.25 mm, (**f**) quantify the percentage of fibrotic images for each treatment group.

This study aims to determine the presence of collagen fibers in liver tissue using SHG microscopy. To achieve this goal, the following steps were executed: planning the treatment groups, sacrificing the animals for liver biopsies, analyzing the resulting samples with the second harmonic generation microscope, classifying the images into fibrotic and non-fibrotic categories using machine learning algorithms, and finally, evaluating the percentage risk associated with each treatment (see Figure 1).

### 2.1. Biological Experimental Design

In this study, 24 male Wistar rats were randomly divided into four groups (six animals for each group) according to dietary treatment: control (C), arsenic (A), sucrose (S), and combined arsenic–sucrose (AS). Each group was maintained under controlled environmental conditions with free access to food and water, after which liver tissues were collected for SHG microscopy analysis. All procedures were conducted in accordance with institutional ethical guidelines for animal research.

#### 2.1.1. Metabolic Syndrome Model in Rat

In line with the principles for reducing the number of animals used in research, we performed the experiments for this work using liver samples from the same rat cohort used in our previous work. We aimed to characterize the alterations induced by arsenic and sucrose, leading to physiopathological changes related to metabolic syndrome [9]. Briefly, we used young adult male Wistar rats (250–280 g; approximately eight weeks of age) obtained from the local facility at Instituto de Fisiología Celular (IFC), UNAM. Rats were randomly separated into four groups (C, S, A, and AS groups), which received these treatments for eight weeks. Control (C, maintained ad libitum with tap water), sucrose (S, treated with 20% (*w*/*v*) sucrose in drinking water ad libitum), arsenic (A, treated with 50 ppm of sodium arsenite in drinking water ad libitum), and arsenic + sucrose (A + S, treated with 50 ppm of sodium arsenite + 20% sucrose in drinking water ad libitum). All animals were fed ad libitum with standard rat chow (Lab Diet 5001, USA) composed of 28.5% protein, 13.5% fat, and 58% carbohydrates. Animals were housed in a 12 h light/dark cycle at 20–23 °C and 40% relative humidity. The experimental rats had an average daily water intake of 15 mL, resulting in a total arsenic exposure of 750 µg per day. While the dosage administered to the animal models is high relative to standard daily intakes, it is comparable to the levels consumed by human populations with chronic exposure to high arsenic toxicity.

#### 2.1.2. Histology and Oil Red O Staining

In this work, we used samples obtained from the right ventral lobe of the liver from the animals sacrificed after overnight fasting (6 animals in each treatment). Liver sections were embedded in Tissue-Tek O.C.T. mounting medium, quickly frozen in isopentane on liquid nitrogen, and stored at −70 °C. To analyze SHG images of collagen fibers, we used liver histological sections with a thickness of 10 µm. Histological sections with a thickness of 3 µm were prepared, fixed with 4% paraformaldehyde, and stained with oil red O (ORO) or hematoxylin & eosin. Independently, liver sections were fixed with 4% paraformaldehyde and embedded in paraffin to obtain histological sections of 7 µm thickness for Masson’s trichrome staining. Images were obtained using a DM500 compound microscope (Leica Microsystems, Germany) with 10×, 40×, and 100× objectives. The percentage of the area stained with ORO was measured using the Fiji software 2.17.0 [33].

### 2.2. Experiment

**Figure 2:**
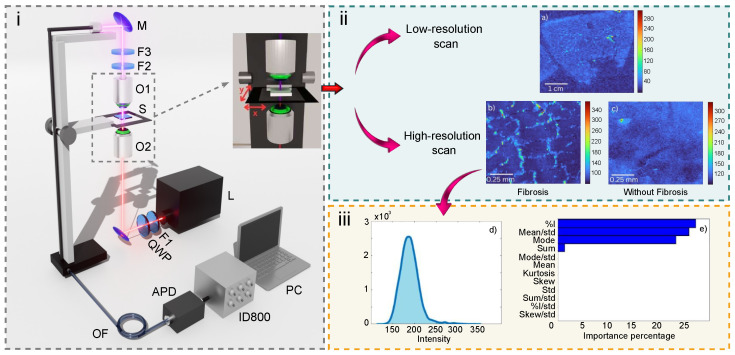
(**i**) The experimental setup for acquiring second harmonic generation (SHG) images comprises a pulsed laser (L), a mirror (M), a 2D scanning station, spectral filters (F1, F2, and F3), objectives (O1 and O2), an optical fiber (OF), a quarter-wave plate (QWP), and an APD. (**ii**) Sample analysis involved two scanning modalities: (**a**) a low-resolution scan providing a whole-sample (S) overview, and (**b**,**c**) high-resolution scans of selected regions for detailed analysis. Image (**b**) shows a sample with fibrosis, whereas image (**c**) shows a sample without fibrosis. (**iii**) The data analysis process consisted of (**d**) calculating probability distributions, and (**e**) determining the relative importance of the first four statistical moments, as well as the statistical mode, the total intensity sum, and the percentage of pixels exceeding 15% of the noise threshold using Random Forest model.

The next step in the methodology is the optical experimental setup. The experimental setup used for the generation of SHG images of collagen fibers is shown in Figure 2(i). A femtosecond Ti:Sapphire laser system (L), tuned to a central wavelength of 810 nm with a bandwidth of 20 nm, operating at a repetition rate of 90 MHz, and delivering an average power of 220 mW, was utilized as the pump. The laser beam was spectrally filtered using a bandpass filter centered at 800 nm with a transmission width of 25 nm (F1, FF01-800/25, Semrock, USA) and its polarization was adjusted to circular by transmission through a quarter-wave plate (QWP), enabling orientation-independent analysis.

The circularly polarized beam was focused onto a rat liver tissue sample, encapsulated within a microscope slide, using an objective lens with a numerical aperture (NA) of 0.65. At the focal point within the tissue, SHG signals were generated. The sample was positioned on a high-speed motorized stage (MLS203-1, Thorlabs, USA) providing two-axis scanning, up to 250 mm/s, 0.25 µm minimum step resolution, and 3 µm positional accuracy.

The SHG emission was collected by a second objective lens with the same numerical aperture as the focusing objective. The emitted light was then spectrally filtered using a low-pass filter (F2, FGB39, Thorlabs) and a 400 nm bandpass filter with a 40 nm bandwidth (F3, FF01-400/40, Semrock) to eliminate any residual pump laser light. Finally, the filtered SHG signal was directed toward the detection system. This last system uses an optical fiber to guide the SHG signal to an avalanche photodiode (APD), where each detected incident photon triggers an electrical pulse. These pulses are transmitted via cable to an ID800 counter (IDQuantique, USA), which registers the number of detected pulses within a user-defined time window, set to 100 picoseconds for this study. The recorded pulse counts are then used to reconstruct the SHG images computationally.

As mentioned above, four types of rat liver tissue samples were analyzed, differentiated by the diet administered to the animals. The groups included: control diet, high-sucrose diet, high-arsenic diet, and a combined arsenic–sucrose diet, each comprising samples from six distinct individuals. Each tissue sample, with a thickness of 10 µm and transverse dimensions of 1 cm × 1 cm, initially underwent a low-resolution scan with a 100 µm step size to obtain a whole-sample overview, as shown in Figure 2(ii-a). Subsequently, ten 1 mm × 1 mm regions were randomly selected, excluding imperfections and portal areas, for high-resolution scanning at 10 µm step sizes. An example of these regions is displayed in Figure 2(ii-b). In accordance with our experimental design, we generated a total of 60 high-resolution scans per sample group.

### 2.3. Machine Learning

#### 2.3.1. Feature Selection

Our dataset of SHG images derived from collagen fibers consists of 240 images, comprising images including both fibrosis-positive and fibrosis-negative images. Each image is represented as a 100 × 100 pixel matrix, where each pixel in this matrix corresponds to an intensity value. Figure 2(ii-b,ii-c) show examples of samples with and without the presence of fibrosis, respectively.

A square window of size 20 × 20 pixels was systematically displaced across the image with a stride of one pixel in both spatial directions. For each window position, the pixel intensities were flattened into a one-dimensional vector and used to compute statistical properties of each image in the dataset. Figure 2(ii-b) shows an example of an image processed with the sliding window. For each submatrix, an intensity probability distribution was computed based on the pixel intensity values. Figure 2(iii-d) illustrates the probability distribution obtained from this analysis. From this distribution, the first four statistical moments (mean, standard deviation, skewness, and kurtosis), as well as the statistical mode, total intensity sum, and the percentage of pixels exceeding 15% of the noise threshold, were obtained.

Using statistical features extracted from 20 × 20 pixel images, we trained a random forest algorithm to identify the key parameters that distinguish fibrosis-positive from fibrosis-negative images, as labeled by a specialist. As a supervised machine learning algorithm, random forest facilitates feature selection by prioritizing the parameters most relevant to the classification task. Notably, the percentage of pixels with intensities exceeding 15% of the noise threshold, the ratio of the mean to the standard deviation (Mean/Std), the statistical mode (specifically reflecting image noise), and the total sum of pixel intensities—parameters collectively representing the image intensity characteristics—were identified as essential for achieving high classification accuracy. The outcomes of the random forest algorithm are illustrated in Figure 2(iii-e).

#### 2.3.2. Unsupervised Learning

To validate specialist annotations and reduce human bias, we implemented an unsupervised validation step. Specifically, we incorporated an unsupervised learning approach to perform an initial classification of the images based on intricate mathematical and statistical features that are difficult to discern visually. Unsupervised learning, a branch of machine learning, aims to identify patterns within unlabeled data through the analysis of features. A significant application of unsupervised learning is clustering, which organizes data points into groups based on shared characteristics [34]. This technique is often employed as a data preprocessing step before the application of supervised learning algorithms, such as neural networks for classification [34].

**Figure 3:**
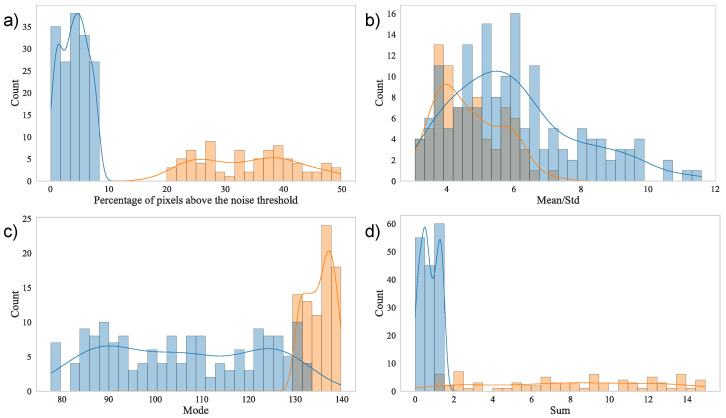
Distribution of (**a**) percentage of pixels above the noise threshold; (**b**) mean/std ratio; (**c**) standard deviation; (**d**) mode and total sum per sliding window. The data in blue corresponds to images without fibers, and the data in orange corresponds to images with fibers. Gray bars indicate overlap between the two distributions.

We implemented the K-Means clustering algorithm, which is one of the most popular and effective clustering methods in the data science community [35]. The K-Means algorithm operates as follows: First, each data point is placed in an $R^{n}$-dimensional Euclidean space, with its coordinates determined by the computed features. In this study, we computed four relevant mathematical features for each image: the arithmetic mean, standard deviation, mode, and the percentage of pixels with an intensity greater than the noise threshold. The noise threshold is defined as one-tenth of the maximum amplitude of the Fourier-transformed signal. These features, displayed in Figure 3, are presented as histograms, with each class (fibrosis and no fibrosis) represented separately.

**Figure 4:**
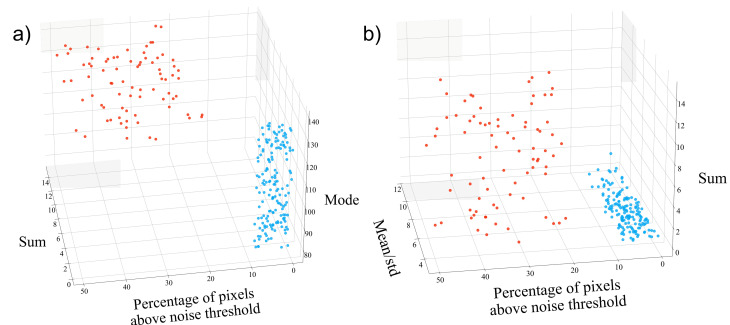
K-means clustering projected onto feature subspaces: (**a**) projection without mean/std; (**b**) projection without Sum. The data in blue corresponds to images without fibers, and the data in red corresponds to images with fibers.

The K-means algorithm is initialized with the number of clusters into which the data will be separated. In this case, we set the algorithm to create two clusters, corresponding to the two possible classifications based on the presence of fibers. Initially, the algorithm selects two random points as centroids in Euclidean space. For each point in the space, the distance to each centroid is computed, and the point is assigned to the nearest centroid. This process is iterated until two distinct clusters are formed. In each iteration, new centroids are recalculated as the average of the points within the clusters formed in the previous step, and points are reassigned to the closest centroid. The algorithm continues until either the maximum number of iterations (set to 500 for this study) is reached or the centroids no longer change significantly. The final clusters are those that remain after the algorithm converges. Figure 4 presents a three-dimensional scatter plot of the features extracted from the second harmonic generated images, classified by the K-means algorithm, aligning with the previous human classification.

#### 2.3.3. Supervised Machine Learning Image Classification

**Figure 5:**
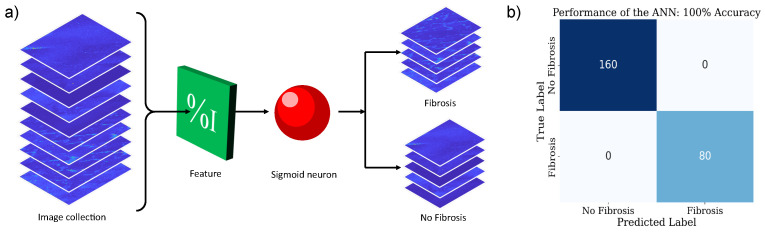
(**a**) Model architecture and feature flow; (**b**) aggregated confusion matrix over cross-validation folds.

To develop a classification algorithm tailored for detecting fibrosis in rat liver samples from SHG images, we implemented a binary classification artificial neural network (ANN). Given that the feature shown in Figure 3a exhibits linearly separable behavior, we used it to enable the classification task. The proposed ANN architecture consists of a single sigmoid neuron. This neuron generates a value between 0 and 1, representing the probability that the image contains fibrosis. Outputs greater than 0.5 are classified as fibrosis-positive. Despite its simplicity, this algorithm offers a robust and reliable classification method. This design choice was motivated by the fact that the extracted features are linearly separable in the transformed feature space, allowing accurate classification with a linear decision boundary. Under these conditions, increasing model complexity does not yield a significant performance improvement and may instead introduce unnecessary computational overhead and a higher risk of overfitting. More complex architectures, such as convolutional neural networks, are particularly advantageous for high-dimensional structured inputs requiring hierarchical feature extraction, which is not the case for the physically meaningful, pre-engineered features used in this study. The model architecture is illustrated in Figure 5a.

The model was trained using a dataset of 240 images, of which 160 depict healthy liver tissue and 80 exhibit hepatic fibrosis. It is important to note that all images were first classified by a human specialist and subsequently validated using the K-Means unsupervised learning algorithm. The learning rate was set to 0.05, a hyperparameter that controls the speed of convergence, and was manually tuned. For optimization, we employed the Adam algorithm due to its computational efficiency and ease of implementation [36]. Model training was performed using the binary cross-entropy loss function (see Equation (1)), which guided the algorithm to minimize classification error. Because of its effectiveness, this loss function is widely used in binary classification tasks.

(1) $$\mathrm{Binary}\;\mathrm{Cross}\text{-}\mathrm{Entropy}=-\frac{1}{n}\sum_{i=1}^{n}\left[y_{i}\log\left(p_{i}\right)+\left(1-y_{i}\right)\log\left(1-p_{i}\right)\right],$$

where *n* is the number of data points, $y_{i}$ represents the true label (0 or 1) for each sample, and $p_{i}$ is the predicted probability that the sample belongs to class 1 [37]. We implemented the K-fold cross-validation method to validate the model. Specifically, we used 20-fold cross-validation to estimate generalization performance. This method involves splitting the data into a specified number of clusters, also referred to as folds. During the first iteration, one fold is excluded, and the remaining folds are used to train the model. The model is then validated using the remaining fold. In the subsequent iteration, the next fold is excluded, and the process continues until all folds have been used for validation. Once all iterations are completed, the model’s performance across all folds is averaged [38].

## 3. Results

Hepatic steatosis and fibrosis were analyzed in liver tissue sections from the C, S, A, and AS groups (see Section 2). Livers from male Wistar rats treated with sucrose and arsenic–sucrose showed increased lipid accumulation compared to C and A groups. The percentage lipid content measured by ORO was 2.5 ± 0.3%, 4.1 ± 0.4%, 3.1 ± 0.4%, and 6.7 ± 1.27% for C, S, A, and AS, respectively.

In addition, Masson’s trichrome staining revealed extensive areas of fibrosis in liver tissues from the S and AS groups, whereas the C and A groups showed minimal collagen fiber areas. These observations, made with standard histological techniques, are consistent with the subsequent quantitative analyses described in this study.

By applying the K-means algorithm, SHG microscopy images were successfully separated into two categories: those exhibiting collagen fibers and those lacking them. As mentioned above, these labeled classes were subsequently used to train a neural network for classification. The algorithm demonstrated excellent performance in the classification task, achieving results comparable to state-of-the-art models with greater computational complexity. For each K-Fold iteration, a confusion matrix was generated. These matrices were then aggregated to create a final confusion matrix (which is presented in Figure 5b). The algorithm achieved perfect classification accuracy, with F1 score, sensitivity, and specificity all equal to 1.0, indicating flawless performance in identifying collagen fibers without false positives or false negatives.

Furthermore, categorized images were mapped to their corresponding dietary groups, allowing quantification of collagen presence per group. The percentage of images containing collagen fibers for each group is shown in Table 1. These results confirm the robustness of the machine learning pipeline for accurately detecting and quantifying collagen fiber remodeling in liver tissues.

Quantitative analysis revealed significant differences in collagen fiber percentages across the experimental groups. Collagen fibers were sparsely detected in control (C) samples, increased in arsenic (A) and sucrose (S) groups, and were most abundant in the arsenic–sucrose (AS) group, where fibers appeared in over half of the analyzed images. These results highlight a progressive accumulation of collagen associated with dietary interventions, supporting the observed trends in fibrosis risk.

**Figure 6:**
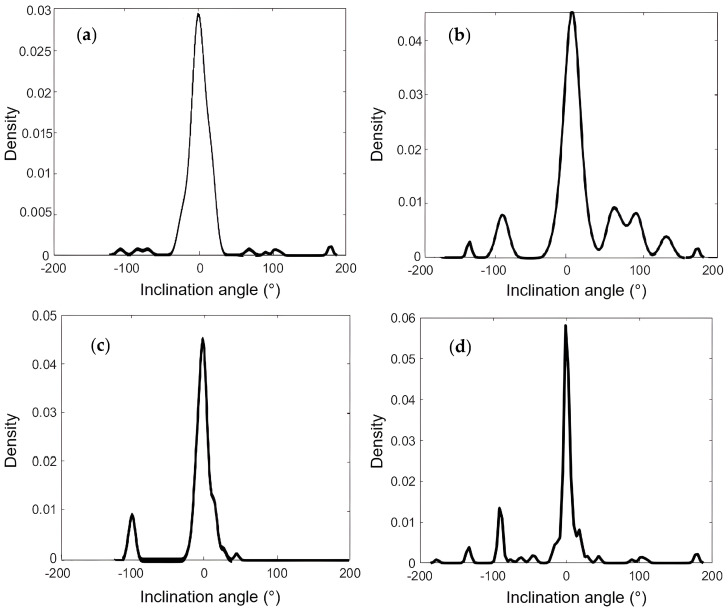
Angularprobability distribution in the samples of (**a**) Group C, (**b**) Group A, (**c**) Group S, and (**d**) Group AS.

The organization of collagen fibers in liver tissue provides an important indicator of tissue damage. In the early stages of liver fibrosis, collagen fibers are randomly distributed, whereas as the disease progresses, the collagen fibers proliferate, enlarge, and form distinct patterns. Comparative analysis revealed significant differences in collagen fiber organization and distribution among groups subjected to different dietary regimens. Fiber orientation was quantified using the Hough transform. Then, one fiber was randomly selected as a reference, and the orientation angles of the remaining fibers were measured relative to it. Interfibrillar collagen orientation angles were quantified from the images containing fibers, and an angular distribution histogram was generated, as shown in Figure 6. Variations in histogram distribution were observed among the groups, with group AS exhibiting reduced angular dispersion, whereas the control group showed broader dispersion (see Table 2). These results suggest that group C samples exhibit less tissue damage, while group AS samples, characterized by more organized collagen fibers, indicate a more advanced stage of fibrosis.

## 4. Discussion

We decided to focus this study on male Wistar rats because, on one hand, males develop more hyperinsulinemia and insulin resistance in metabolic syndrome, and on the other hand, several reports indicate that males are more prone to develop liver fibrosis [39]. Moreover, estrogen and progesterone may play a protective role in the development or progression of fibrosis [40,41,42]. Furthermore, metabolism is sex-dependent in rats, a factor important for the response to different dietary interventions [43,44,45]. Also, female and male rats respond differently to pollutants [46], a factor relevant in one of our study groups.

In Group A, the probability of liver fibrosis was 24%, higher than in the control group but still relatively low, suggesting that chronic exposure to low levels of dietary arsenic poses a moderate risk for fibrosis development. Nevertheless, further studies are needed to assess the long-term effects of low-dose arsenic consumption. In contrast, Group S exhibited a nearly 50% probability of fibrosis, indicating a strong association between a sugar-rich diet and potential liver damage. These results indicate that high dietary sugar significantly increases fibrosis risk, although it does not necessarily lead to fibrosis in all cases. Notably, the AS group showed a markedly higher probability of liver fibrosis, consistent with the trends observed in the other groups. This finding suggests that arsenic adds to the harmful effects of sucrose, resulting in more pronounced liver tissue damage when both dietary factors are combined.

Remarkably, the angular distribution of collagen fibers was widest in group C samples and narrowest in the arsenic–sucrose (AS) group, with Group A and S showing intermediate values, demonstrating a closer proximity to group C. This indicates a more disordered collagen fiber arrangement within group C livers, whereas group AS livers exhibit highly organized collagen structures. The probability of liver fibrosis was less than 10% in the control group, reflecting the absence of liver-toxic dietary agents, although it is important to note that this risk is not zero, due to factors beyond dietary intake, such as genetic predispositions.

Second harmonic generation is a powerful technique for visualizing collagen fibers, enabling the straightforward application of machine learning algorithms for image classification without the need for additional image processing. The effective classification of images with and without collagen fibers was achieved by combining SHG imaging, statistical analysis, an unsupervised machine learning algorithm, and a binary classifier based on neural networks. Our algorithm achieved exceptional performance in the classification task, attaining an overall high accuracy despite its simplicity.

Based on these classifications, fibrosis risk probabilities were estimated for each dietary group. Additionally, an analysis of collagen fiber orientations revealed a significantly narrower angular distribution in the AS group samples, indicating more organized fiber structures compared to other groups. Overall, these results suggest that dietary sucrose and/or arsenic increase the risk of liver fibrosis, with a notably higher risk when both are present, and highlight the potential of combining SHG microscopy with machine learning for quantitative assessment of collagen remodeling and early fibrosis detection.

SHG microscopy is a coherent optical imaging technique for which the spatial resolution is fundamentally constrained by the diffraction limit. Collagen fibers typically range from 100 to 500 nm in diameter and are commonly organized into dense bundles [47]. The structural patterns visualized in SHG microscopy arise from the coherent summation of signals from multiple fibers [48]. Consequently, the image reflects the spatial distribution of fiber clusters rather than the physical dimensions of single filaments. This causes the visualized fiber width to be a representation of the diffraction boundary instead of the physical thickness of individual fibers.

In SHG microscopy, the optical signal contributing to images is derived specifically from tissue exhibiting a second-order optical non-linear response, particularly collagen. This is fundamentally different from standard microscopy used in histology (with techniques such as Masson). SHG offers significant advantages: it allows the visualization of collagen fibers in the absence of other types of tissue, image deeper into the sample, does not require chemical processing, prevents phototoxicity, can image thicker samples in 3D without sectioning or staining, and enables the visualization of the collagen architecture in optically dense tissues that are otherwise difficult to analyze [49]. The synergy between SHG imaging and ML offers a promising path for future clinical applications.

The potential of using SHG microscopy and machine learning in clinical diagnosis is automation, as it allows for the analysis of large amounts of data in a short time. It also increases precision because collagen is the only protein that produces SHG, making it easy to identify diseases in their early stages. This reduces diagnosis time, which is key to successful treatment [50]. The main challenges are the use of sensitive information and avoiding generalities that lead to errors [51]. Some databases might not be useful for the whole population because, for example, the structure of the liver changes with age.

## 5. Conclusions

This work demonstrates that a diet rich in sucrose, particularly in combination with arsenic, constitutes a significant risk factor for liver collagen fiber remodeling. Machine learning accurately classified SHG microscopy images based on the presence of collagen fibers in the liver tissues. Fibrosis risk was low (<10%) in controls, increasing to 24% in a diet with arsenic, 40% with sucrose, and 62% when both were combined. Collagen fiber orientation became progressively narrower with sucrose and arsenic–sucrose diets, reflecting structural changes predictive of fibrosis severity. Four statistical features identified by random forest classification enabled precise collagen detection, revealing that sucrose effects are amplified by the presence of arsenic (even at low concentrations). Thus, combining SHG microscopy with machine learning provides a robust framework for early detection and quantitative assessment of diet-induced liver fibrosis.

## Figures and Tables

**Table 1:** Percentage of images containing visible collagen fibers by sample type. Values reported as mean ± SD.

Group	Percentage of Images with Fibers
Control group	8.0 ± 3.0%
Arsenic group	24 ± 5.7%
Sucrose group	40 ± 5.9%
Arsenic–sucrose group	62 ± 18%

**Table 2:** Angular distribution width (degrees) of collagen fiber orientations for each sample group.

Group	Distribution Width (°)
Control group	26
Arsenic group	24
Sucrose group	16
Arsenic–sucrose group	2.8

## Data Availability

The data used for this work are available at https://drive.google.com/drive/folders/1Poz27xrxRX_3lxxLTom1r8tU__z8L53P?usp=drive_link (accessed on 1 December 2025). All Python 3.12.10 scripts used in this study can be found at https://github.com/CFATA-AI/Machine-Learning-on-Second-Harmonic-Generation-Microscopy-Images (accessed on 1 December 2025).

## Abbreviations

The following abbreviations are used in this manuscript:

NAFLD	Non-alcoholic fatty liver disease
SHG	Second-harmonic generation
BMI	Body mass index
